# Bridging the proton gap: A proton therapy consultation service for Canadian radiation oncologists^☆^

**DOI:** 10.1016/j.tipsro.2025.100320

**Published:** 2025-06-11

**Authors:** Inhwa Kim, Amy Parent, Michael Holwell, Tim Craig, Patricia Lindsay, Hillary Le, Yat Tsang, Perry B. Johnson, Danny J. Indelicato, Fei-Fei Liu, Derek S. Tsang

**Affiliations:** a Department of Radiation Oncology, University of Toronto, Toronto, Ontario, Canada; b Radiation Medicine Program, Princess Margaret Cancer Centre, University Health Network, Toronto, Ontario, Canada; c Department of Radiation Oncology, University of Florida College of Medicine, Jacksonville, FL, USA

**Keywords:** Proton therapy, Radiation dosimetry, Radiotherapy planning, computer-assisted

## Abstract

**Purpose:**

Canada is the only G7 country without a clinical proton beam therapy (PBT) facility. Patients can access publicly funded PBT through provincial out-of-country approval programs. We created a consultation service to inform health care providers regarding the potential dosimetric benefits of PBT.

**Materials and Methods:**

We retrospectively reviewed patients referred to our proton consultation service from June 2020 to December 2024. Pencil beam scanning proton plans were generated in the RayStation treatment planning system. Key dosimetric parameters were evaluated using Wilcoxon matched-pairs signed rank test.

**Results:**

A total of 55 patients were referred to our service, and 49 had evaluable plans. Patients were referred from 6 provinces and 11 institutions across Canada. The median age at referral was 36 (range, 3–74 years), and 29 % of patients were pediatric. The most common reasons for referral were young age (33 %) and proximity of tumour to OARs (37 %). Target coverage was similar in both photon and proton plans. Cranial (n = 19) and thoracic (n = 7) plans showed a statistically significant reduction in mean brain dose (p < 0.001), mean brain dose minus PTV (p < 0.001), brain D50% (p < 0.001), mean lung dose (p = 0.02), lung V50% (p = 0.02), and lung D50% (p = 0.02) with proton therapy. Sixteen patients were approved by their provincial Ministry of Health for PBT in the United States.

**Conclusion:**

Our proton consultation program provides an important service by identifying patients where PBT offers a dosimetric advantage over photon therapy. Following this personalized, data-informed approach, nearly a third of referred patients were approved to go to the US for PBT.

## Highlights

• Canada is the only G7 country without a clinical proton beam therapy facility.
• This is the first comparative proton consultation service in Canada.
• Minimizing radiation exposure to normal tissues reduces acute and late toxicities.
• Common reasons for referral were young age and proximity of tumor to normal organs.
• Almost a third of referred patients ultimately went on to have out-of-country PBT.

## Introduction

Proton beam therapy (PBT) delivers highly conformal radiotherapy to tumour targets while sparing nearby normal tissues to a greater degree than photon therapy. Minimizing radiation exposure to normal tissues is crucial for pediatric and adolescent/young adult (AYA) patients with cancer, as their longer life expectancies increase their risk for late effects associated with radiation [1]. Adult patients may also benefit from PBT because of reduced acute toxicities with radiation [2].

There are currently 137 operating PBT centres in 23 countries as of March 2025 [3]. Canada is the only G7 country without a clinical PBT facility. There have been public announcements by various provinces committing to bringing PBT to Canadians, including Quebec in 2018 [4], Ontario in 2022 [5], and in Alberta 2024 [6], but there is not yet a PBT facility under construction. While Canadian patients can access publicly funded PBT through provincial out-of-country approval programs, there are barriers to accessing care, including varying provincial eligibility criteria, administrative delays, and indirect costs associated with travel [1,7,8].

Our institution created a proton therapy consultation service in June 2020, which allows any radiation oncologist in Canada to request the creation of a personalized proton plan for their patient. The goal of this service was to inform health care providers regarding the potential dosimetric benefits of proton therapy compared to standard photon treatments, especially in cases where the benefit of protons was unclear. This service aligns with the pan-Canadian consensus recommendations for PBT access in Canada [9]. The objective of this study was to retrospectively review all patients referred to our institution’s proton consultation service.

## Materials and methods

### Study design

We completed a retrospective review of all patients referred to our proton consultation service from its inception in June 2020 to December 2024. Patients were identified from a prospectively maintained administrative database. Additional data was obtained through retrospective review of available patient charts.

All available photon and proton plans were reviewed on RayStation treatment planning system (TPS, version 10, RaySearch Laboratories, Stockholm, Sweden). Clinical target volume coverage by 95 % of the prescribed dose was collected from both photon and proton plans. Given the wide variety of tumour sites included in our consultation service, focused dosimetric parameters were collected for cranial and thoracic targets. These targets were specifically chosen for consistency in OARs for comparison, as targets in the neck, abdomen, and pelvis varied greatly in location with differences in affected OARs. This study was reviewed by the research ethics board of the University Health Network (CAPCR ID 23-5325).

### Proton consultation service

Radiation oncologists in Canada were able to refer any patients undergoing curative intent radiotherapy (http://www.protonsatuhn.ca). Those requiring urgent commencement of treatment were excluded due to the anticipated delays of application for PBT approval and travel out of the country. Referring physicians were asked to provide an RT-DICOM data set with contoured OARs, GTV, CTV, and PTV. It was also recommended that they provide photon plans, but they could also be generated by our institution on request.

A team of dosimetrists, physicists, and radiation oncologists generated a proton plan. Pencil beam scanning was used with a Monte Carlo dose computation engine. A commissioned proton beam model was used from an operating US-based proton centre, with permission. Robust optimization on the CTV was done [10], accounting for patient displacement and density uncertainty based on the tumour site being treated. For example, brain cases were planned to be robust to 3 mm displacements and 3.5 % range uncertainty [11], while extracranial sites were planned considering 5 mm displacements (consistent with photon PTV margins). Thoracic targets were planned with 5 % range uncertainty. A range shifter was used for any beam with a superficial target [11]. All reported proton doses are in cobalt gray equivalents, accounting for relative biological effectiveness of 1.1, which permits direct comparison with photon doses.

The referring physician was provided with a written report and electronic imaging & dose data in RT-DICOM format, including key dosimetric parameters and dose-volume histograms, within 5 to 7 business days. Ultimately, the referring physician was responsible for submitting an out-of-country proton beam therapy application to their respective provincial Ministry of Health, if PBT was deemed to provide a dosimetric and expected clinical benefit.

### Statistical analysis

Baseline characteristics of patients, tumours, and referring institutions were reported descriptively. For patients with cranial and thoracic targets, key dosimetric parameters of both photon and proton plans were evaluated using Wilcoxon matched-pairs signed rank test due to small sample sizes. Statistical analysis was completed using GraphPad Prism 10 software.

## Results

A total of 55 patients were referred to our proton consultation service, and 49 patients had analyzable photon and proton plans. Of the 6 patients that were excluded, 3 patients had incurable disease, 1 patient had significant spinal hardware complicating proton therapy, and 2 patients did not have a comparative photon plan accessible for analysis. The characteristics of the remaining 49 patients are presented in Table 1. The median patient age at referral was 36 (range, 3–74 years), and 29 % of patients were under the age of 18. Patients were referred from 6 provinces and 11 institutions across Canada. Twenty patients (41 %) were referred from outside our institution. The most common reason for referral was for young age (33 %) and proximity of tumour to OARs (37 %); 14 % of referrals were patient-directed to the patient’s primary oncologist, who then requested the referral. The wide range of histological diagnoses of patient tumours, categorized by anatomical location, are listed in Table 2.

**Table 1:** Baseline characteristics of patients (N = 49 patients).

Characteristic	Value (%)
Median (IQR) age at referral, years	36 (38)
0–18	14 (29 %)
19–64	26 (53 %)
>65	8 (16 %)
Unknown	1 (2 %)
Sex	
Male	20 (41 %)
Female	29 (59 %)
Province of residence	
Ontario	40 (82 %)
Quebec	5 (10 %)
British Columbia	1 (2 %)
Saskatchewan	1 (2 %)
Nova Scotia	1 (2 %)
Prince Edward Island	1 (2 %)
Reason for referral	
Proximity to OARs	18 (37 %)
Young age	16 (33 %)
Patient preference	7 (14 %)
Re-irradiation	4 (8 %)
Second opinion	1 (2 %)
Not specified	3 (6 %)

IQR, Interquartile range; OAR, organs-at-risk.

**Table 2:** Histology of patient tumours categorized by anatomical location (N = 49 patients).

Characteristic	Value (%)
**Cranium**	**20 (41 %)**
Meningioma	6 (12 %)
Chordoma	3 (6 %)
Astrocytoma	2 (4 %)
Orbital rhabdomyosarcoma	2 (4 %)
Medulloblastoma	1 (2 %)
Neuroblastoma	1 (2 %)
Ewing sarcoma	1 (2 %)
Solitary fibrous tumour	1 (2 %)
Hemangiopericytoma	1 (2 %)
Optic pathway glioma	1 (2 %)
Non-germinomatous germ cell tumour	1 (2 %)
**Head and neck**	**13 (27 %)**
Nasopharynx carcinoma	4 (8 %)
Paraganglioma	3 (6 %)
Parotid adenocarcinoma	1 (2 %)
Mucoepidermoid carcinoma	1 (2 %)
Extra-renal rhabdoid tumour	1 (2 %)
Ewing sarcoma	1 (2 %)
Malignant peripheral nerve sheath tumour*	1 (2 %)
Adenoid cystic carcinoma	1 (2 %)
**Thorax**	**6 (12 %)**
Non-small cell lung cancer	2 (4 %)
Diffuse large B-cell lymphoma	1 (2 %)
Ewing sarcoma	1 (2 %)
Invasive ductal carcinoma	1 (2 %)
Thymoma	1 (2 %)
**Abdomen/Pelvis**	**10 (20 %)**
Sacral chordoma	4 (8 %)
Nodal metastasis (gastric adenocarcinoma)	1 (2 %)
Rectal adenocarcinoma	1 (2 %)
Renal Ewing sarcoma	1 (2 %)
Sacral solitary fibrous tumour	1 (2 %)
Sacral Ewing sarcoma	1 (2 %)
Rhadomyosarcoma	1 (2 %)

*Referred to our service with diagnosis of MPNST, though pathology review later revealed melanoma for which this patient received immunotherapy.

**Fig. 1:**
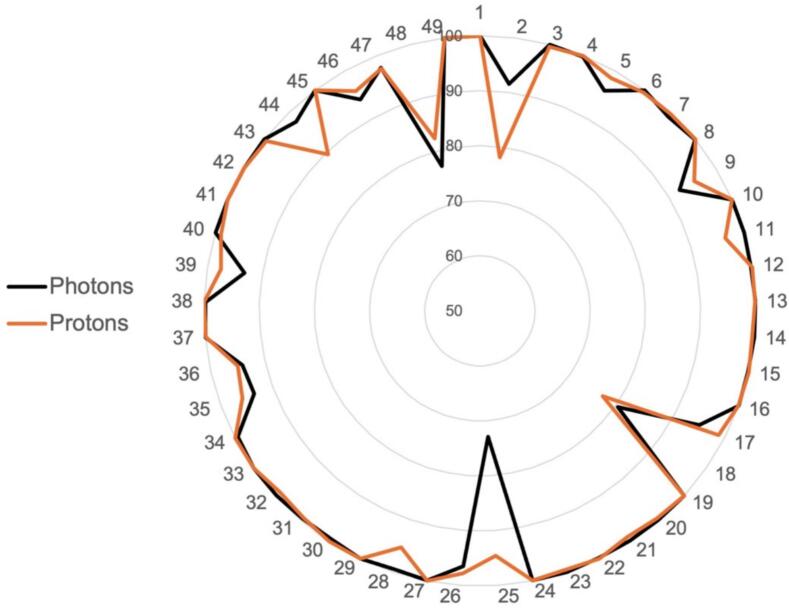
Clinical target volume coverage by 95 % of the prescribed dose in both photon and proton plans for 49 patients. Points closer to the outermost circle represent better tumour coverage.

**Fig. 2:**
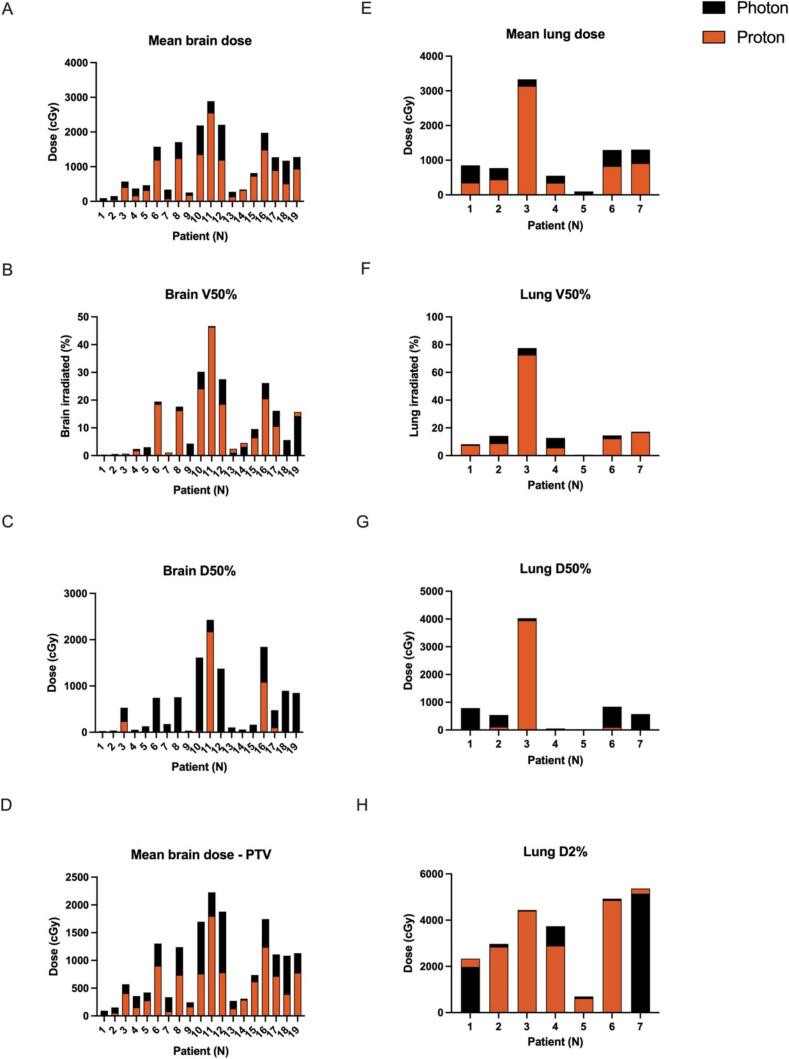
(A-D) Mean brain dose, mean brain dose minus PTV, brain V50%, and brain D50% for 19 patients with cranial targets in both photon and proton plans. (E-H) Mean lung dose, lung V50%, lung D50%, and lung D2% for 7 patients with thoracic targets in both photon and proton plans.

Clinical target volume coverage by 95 % of the prescribed dose was similar in both photon and proton plans (CTV V95% photon 97 %, proton 98 %; p = 0.71) as shown in Fig. 1. For the 19 patients with cranial targets, there was a statistically significant reduction in the mean brain dose (photon 1049 cGy, proton 725 cGy; p < 0.001), mean brain dose minus PTV (photon 676 cGy, proton 480 cGy; p < 0.001), and brain D50% (photon 647 cGy, proton 199 cGy; p < 0.001). There was no difference in brain V50% (p = 0.28). For the 7 patients with thoracic targets, there was a statistically significant reduction in the mean lung dose (photon 1172 cGy, proton 864 cGy; p = 0.02), lung V50% (photon 21 %, proton 18 %; p = 0.02), and lung D50% (photon 978 cGy, proton 603 cGy; p = 0.02) with proton therapy. There was no difference in lung D2% (p = 0.69). These brain and lung dosimetric parameters are shown in Fig. 2A–H. Of note, 1 patient with medulloblastoma treated with craniospinal irradiation was evaluated as a thoracic target for the spinal portion of treatment.

In total, 16 patients were approved by their respective provincial Ministry of Health for out-of-country proton beam therapy, and 14 patients received publicly funded care in the United States. Two patients did not ultimately go on to receive proton therapy due to patient preference for surgical treatment options and due to delays associated with out-of-country health care. One additional patient was not approved for proton therapy, but they chose to fund their own proton therapy in India for the treatment of their solitary fibrous tumour. The diagnoses of 16 patients approved for PBT included 6 cranial targets (neuroblastoma, meningioma, Ewing sarcoma, orbital rhabdomyosarcoma, astrocytoma, non-germinomatous germ cell tumour), 3 thoracic targets (medulloblastoma, invasive ductal carcinoma, non-small cell lung cancer), and 7 others (Ewing sarcoma, mucoepidermoid carcinoma, sacral chordoma, rhabdomyosarcoma, nasopharyngeal carcinoma, paraganglioma).

**Fig. 3:**
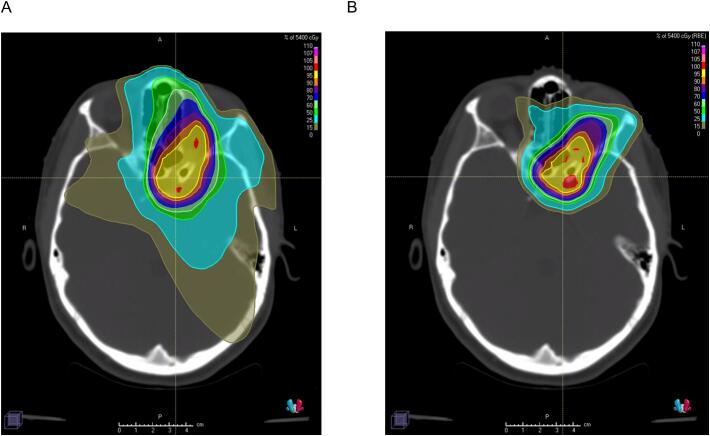
19-year-old woman with a progressive left optic nerve sheath meningioma with orbital apex, intracranial, and suprasellar involvement. The provincial Ministry of Health requested a comparative plan for the planned treatment of 5400 cGy in 30 fractions. (A) Photon plan; CTV V95 100 %, mean brain dose 371 cGy, brain V50 2.4 %, brain D50 53 cGy. (B) Proton plan; CTV V95 99.98 %, mean brain dose 166 cGy, brain V50 1.86 %, brain D50 3 cGy. The isodose line legend is shown in the top right of each panel.

Fig. 3 demonstrates a photon and proton plan of a 19-year-old patient with a left optic nerve sheath meningioma treated with 54 Gy in 30 fractions. She was initially declined by the provincial Ministry of Health for out-of-country PBT. The reasons cited for denial were to consider stereotactic radiosurgery (SRS) as an alternative treatment option and to consider comparative planning to evaluate the dosimetric benefit of proton therapy compared to photons. However, this lesion was too large for SRS. Following referral to our consultation service, our conventionally fractionated proton plan demonstrated comparable target volume coverage (CTV V95% photon 100 %, proton 99.98 %) and considerable reduction in mean brain dose (photon 371 cGy, proton 166 cGy). Brain V50 (photon 2.4 %, proton 1.86 %) and brain D50 (photon 53 cGy, proton 3 cGy) were also reduced. This patient’s oncologist was able to appeal the Ministry of Health decision and was, ultimately, approved and received treatment with proton beam therapy in the US.

## Discussion

Pediatric patients with curable, localized malignancies are routinely considered for out-of-country proton therapy [7,9,12]. PBT may not be clearly beneficial for other tumours, and these patients may benefit from comparative planning. Our proton consultation service had a total of 55 referrals across 6 provinces and 11 institutions in Canada. While the most common reasons for referral were proximity of tumour target to OARs and young age, a small number of referrals were directed by patients via request to their primary oncologist, and the patient’s preference for protons. Of the 49 patients included in our analysis, tumour target coverage by both photon and proton plans were similar. Our focused analysis with cranial (n = 19) and thoracic (n = 7) targets demonstrated a statistically significant reduction in brain and lung doses with PBT. Ultimately, 16 patients were approved for out-of-country PBT, and 14 patients followed through with treatment. For the remaining patients, the treating oncologist felt that photon radiation could deliver similar outcomes as PBT. Even though less than one-third of patients with a comparative proton plan created ultimately went for proton therapy, the service helped guide oncologists regarding suitable cases for out-of-country treatment.

This proton consultation service is the first of its kind in Canada. A similar proton consultation service is carried out in Australia by Hu et al. in 2020, as their out-of-country approval program required comparative planning as a pre-requisite for application. The Australian cohort (n = 43) was significantly younger with 72 % of patients being under the age of 18, compared to 29 % in ours. They had 14 patients approved for out-of-country PBT, which is comparable to 16 patients from our cohort [13]. Australia is building their first PBT facility in Adelaide, though the project is currently delayed [3,14,15].

Due to the wide variety of diagnoses, our dosimetric analysis focused on cranial (n = 19) and thoracic (n = 7) targets. For pediatric brain tumours, PBT is associated with lower rates of radiation-induced toxicities, particularly hypothyroidism and neurocognitive function [12], [16], [17], [18]. Studies of adult brain tumours have not demonstrated significant differences in terms of acute toxicities [19], [20], but there have been modelling studies estimating a modest decrease in the risk of neurocognitive impairment on specific psychological tests from 7 % (photons) to 5 % (protons) and some patient-reported outcomes that suggest a reduction in fatigue with PBT [21], [22]. There continues to be a lack of prospective, level 1 evidence comparing PBT to photon therapy in the treatment of brain tumours, but the current evidence suggests that lower doses to cranial structures will translate to a clinical benefit.

The notion of sparing normal tissues from radiation translating directly to a clinical benefit may be specific to disease site. Liao et al. hypothesized that exposing less lung tissue to radiation with PBT compared to photon therapy would reduce toxicity for patients with inoperable NSCLC. After randomizing 181 patients to either PBT or photon therapy, they did not observe any differences in local failure or grade >3 radiation pneumonitis. While they did demonstrate improved dose-volume indices for the heart, this did not render any detectable clinical outcomes, likely due to a short median follow-up of 24.1 months [23]. Furthermore, patients enrolled later in the study and treated with PBT had significantly less pneumonitis, potentially demonstrating a training effect relating to PBT planning skills. As Canada prepares for a potential future PBT facility, programs such as our proton consultation service will help build skilled training capacity for high quality PBT planning.

Our study is limited by a modest number of referrals (n = 55) over a course of 4.5 years, resulting in 16 referred patients being approved for out-of-country PBT. Most of our cohort (82 %) were from the province of Ontario. The Ontario Health Technology Assessment states that a total of 57 patients were approved for out-of-country PBT between 2010 and 2019 [1]. Given that comparative planning is not a pre-requisite for application and not captured by our consultation service, our sample size is not reflective of the provincial population. There are currently no national databases tracking the patients receiving out-of-country PBT [9]. Secondly, we acknowledge that the wide range of diagnoses does not allow for a robust evaluation of every specific disease sites. When a publicly funded PBT facility is built in Canada, a prospective, comprehensive national proton registry should be a requirement to enable comprehensive reporting of utilization across disease sites and to facilitate robust outcomes-based research.

Canadian patients deserve access to PBT without the barriers to accessing care associated with out-of-country travel. Our national proton consultation program provides an important service to Canadian radiation oncologists by informing them of the potential dosimetric benefits of PBT in comparison to photon therapy. Our service was able to contribute to nearly a third of referred patients going to the US for PBT.

## Informed consent

The author(s) confirm that written informed consent has been obtained from the involved patient in Fig. 3, and they have given approval for this information to be published in this research article.

## Declaration of competing interest

The authors declare the following financial interests/personal relationships which may be considered as potential competing interests: The proton therapy consultation service was funded by internal departmental funding with partial support by the Princess Margaret Cancer Foundation. DST is a consultant with Need (https://www.need.ai), unrelated to this work.
